# The Combined Oral Stable Isotope Assessment of Muscle (COSIAM) reveals D-3 creatine derived muscle mass as a standout cross-sectional biomarker of muscle physiology vitality in older age

**DOI:** 10.1007/s11357-022-00541-3

**Published:** 2022-03-18

**Authors:** Jessica Cegielski, Matthew S. Brook, Bethan E. Phillips, Catherine Boereboom, Amanda Gates, John F. R. Gladman, Kenneth Smith, Daniel J. Wilkinson, Philip J. Atherton

**Affiliations:** 1 MRC-Versus Arthritis Centre for Musculoskeletal Ageing Research and NIHR Nottingham BRC, Clinical, Metabolic and Molecular Physiology, School of Medicine, University of Nottingham, Royal Derby Hospital Centre, Uttoxeter Road, Derby, DE22 3DT UK; 2 Nottingham University Hospitals NHS Trust, Nottingham, UK; 3 MRC/Versus Arthritis Centre for Musculoskeletal Ageing Research, Division of Medical Sciences and Graduate Entry Medicine, University of Nottingham, Royal Derby Hospital Centre, Uttoxeter Road, Derby, DE22 3DT UK

**Keywords:** COSIAM, Muscle, DXA

## Abstract

Validated diagnostics of skeletal muscle vitality could benefit clinical and basic science in terms of mechanistic insights and in determining the efficacy of interventions, e.g. exercise/pharmaceuticals/nutrients. We recently developed a Combined Oral Assessment of Muscle (COSIAM) that can be used to simultaneously quantify whole-body muscle mass (WBMM), muscle protein synthesis (MPS) and muscle protein breakdown (MPB). Here, we aimed to establish, in a cross-sectional fashion, links between COSIAM parameters and established aspects of muscle function. We recruited 37 healthy older adults (male (M):female (F) (21/16); 72 ± 5 y)) into a 3-day trial. Subjects consumed D_3_-creatine (D_3_-Cr dilution to assess WBMM), D_2_O (MPS by incorporation of alanine) and D_3_-3-methylhistidine (D_3_-MH dilution to assess MPB). A biopsy at day 3 was used to determine MPS, and blood/urine samples were collected to determine D_3_-Cr/D_3_-MH dilution for WBMM and MPB. Physiological measures of muscle mass (e.g. DXA/ultrasound) and function (e.g. handgrip strength, maximum voluntary contraction (MVC), one-repetition maximum (1-RM)) were ascertained. A stepwise linear regression approach was used to address links between facets of COSIAM (MPS, MPB, WBMM) and muscle physiology. Despite expected differences in muscle mass, there were no significant differences in MPS or MPB between sexes. WBMM as measured using D_3_-Cr positively correlated with DXA-derived lean body mass (LBM) and appendicular LBM (ABLM). Stepwise linear regression was used to assess which combination of MPS, MPB, D_3_-Cr and absolute synthesis rate (ASR) best predicted physiological measures of muscle health in these older adults. D_3_-Cr WBMM alone was the best predictor of handgrip, 1RM and MVC, and outperformed more traditional measures of muscle mass by DXA. The COSIAM approach substantiates D_3_-Cr as a robust biomarker of multiple muscle physiology health biomarkers. Future work using COSIAM should focus upon how and which parameters it can inform upon in relation to disease progression and the efficacy of interventions.

## Introduction

Declines in muscle mass and function are hallmarks of ageing culminating in the manifestation of sarcopenia and frailty [20]. As a result, those living with sarcopenia face an increasing risk of falls, fractures and loss of independence [21]—engendering a major social and economic burden in the face of the sustained spike in longevity across many parts of the world. Muscle mass is metabolically regulated by proteostasis, ultimately reflected in the longer-term balance between muscle protein synthesis (MPS) and muscle protein breakdown (MPB). As such, the measurement of these facets alongside muscle mass itself remains a cornerstone to understanding the basis of age-related muscle loss, but also the efficacy of mitigation strategies, such as exercise [12], nutrition [3, 6] and pharmaceuticals [8]. Finally, iterations of sarcopenia diagnostic criteria, e.g., by the EWGSOP [1], have established an ICD-10 code for sarcopenia, which may encourage the pharmaceutical industry to re-visit the impact of drugs and value of biomarkers relating to this progressive condition.

The principal methods to assess “muscle health” (or vitality) in the literature centre around measures of whole-body or regional muscle mass, muscle function and protein turnover, i.e. MPS and MPB. We recently developed a minimally invasive Combined Oral Stable Isotope Assessment of Muscle (COSIAM) approach where we provide a protocol capable of simultaneous quantification of muscle mass (via a deuterated creatine dilution technique [5, 16, 17]), muscle protein synthesis (via D_2_O [2, 18, 19]) and muscle protein breakdown (via deuterated 3-methylhistidine dilution [5, 14]). The purpose of setting up this approach was to offer researchers a single protocol to determine these variables in older, clinical (vs. healthy) and difficult to study populations, and also in response to candidate interventions. Nonetheless, from a cross-sectional perspective, an important next-step is to assess how COSIAM facets link to broader physiological biomarkers of muscle health (i.e. mass/functional characteristics).

In our previous paper [5], we conducted a pilot study in a small number (*N* = 10) of subjects aimed at validation of the COSIAM approach from a purely technical and logistical perspective. The aim of the present study was to expand this pilot work and determine relationships between COSIAM facets and cross-sectional muscle health parameters in a larger group of older men and women. We sought to determine which facets of COSIAM relate to functional muscle health parameters (e.g. handgrip strength, leg strength, muscle mass by DXA imaging), on a cross-sectional basis in older individuals of both sexes.

## Methods

### Participant recruitment, ethics and study data collection

This work utilises and extends data collected as part of previously published work [5]. This study was reviewed and approved by the University of Nottingham Faculty of Medicine & Health Sciences Research Ethics Committee (A08122015) and was performed in accordance with the Declaration of Helsinki (2013). Thirty-seven healthy older (M:F (21/16); 72 ± 5 y)) adults were recruited. All participants were screened by means of a medical questionnaire, physical examination and resting ECG. Participants were excluded if they had a BMI > 35 kg·m^2^, active cardiovascular disease, cerebrovascular disease, respiratory disease, metabolic disease, inflammatory bowel or renal disease, active malignancy, any musculoskeletal or neurological disorders, any signs of clotting dysfunction, or any recent (within 6 months) steroid treatment, or if on hormone replacement therapy. All participants were informed of the purpose of the study and of all the risks and procedures involved, before providing their written, informed consent to participate.

On the first study day (day 1), participants were asked to attend the unit at 8:30 am for a whole-body DXA scan, having fasted overnight. They next provided a baseline saliva sample (to measure baseline body water enrichment); a baseline blood sample was then taken to measure background blood/plasma alanine labelling prior to D_2_O exposure, and baseline urine sample collected to measure D_3_-creatinine background enrichment. Muscle architecture was measured by ultrasound before performing any muscle function assessments. Participants then performed the Short Physical Performance Battery (SPPB). Leg extensor (unilateral one-repetition maximum (1-RM) and maximal voluntary contraction (MVC) for leg extension) and handgrip strength (using handgrip dynamometer) were also measured. Participants were then given three 50-ml aliquots of 70 atom percent (AP) D_2_O to drink, each taken 25 min apart (to minimise the risk of side effects such as dizziness or nausea that are sometimes reported after D_2_O consumption due to disturbance of specific gravity within the vestibular fluid [10]). Thirty milligrammes D_3_-Cr was included in the final 50-ml aliquot of D_2_O. Two hours after consumption of the final D_2_O aliquot, participants provided another saliva sample, to be used to determine the plateau D_2_O labelling in body water.

Prior to leaving the laboratory, participants were provided with a container to collect all urine for 24 h, a tube for a spot urine sample at 48 h and three tubes for saliva samples, collected at 12, 24 and 48 h after the final D_2_O aliquot was consumed. The urine samples were used to measure D_3_-creatine spill-over, urinary D_3_-creatinine enrichment and the size of the creatine pool, respectively. Saliva was collected to monitor the decay in D_2_O over time to determine precursor alanine labelling. Participants were also given 10 mg of D_3_-3MH dissolved in 50 ml of water to take home and store refrigerated. Participants were instructed to consume the D_3_-3MH immediately 48 h after the urine and saliva samples were collected.

**Fig. 1:**
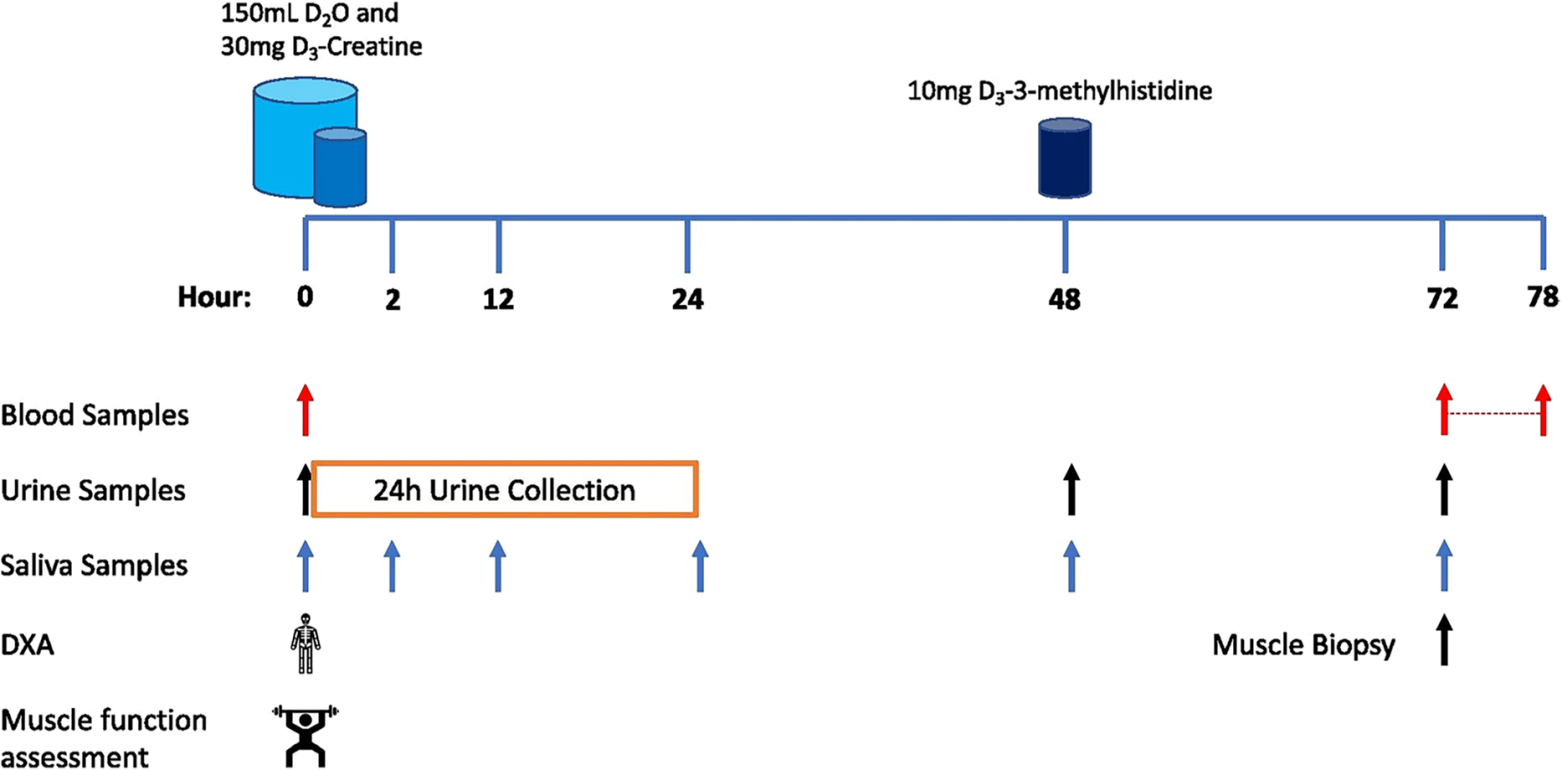
Study schematic highlighting the simplicity of the COSIAM approach for simultaneously determining muscle protein synthesis (MPS), breakdown (MPB) and muscle mass ( taken from Cegielski et al. 2020). Abbreviations: DXA, dual-energy absorptiometry

Participants returned to the laboratory 72 h after consumption of the final D_2_O aliquot which was ~ 20–22 h after the D_3_-3MH consumption, again having fasted overnight. At this visit, hourly bloods were collected over 6 h (between 22 and 28 h after consumption of the D_3_-3MH dose), with further spot urine and saliva samples collected at 72 h after D_3_-3MH consumption. A muscle biopsy from the *m. vastus lateralis* (VL) was also taken at the start of this visit (72 h) under local anaesthetic (1% lidocaine). The Bard micro-needle biopsy technique (50% length of VL, mid-belly) was used for the first 8 participants, with the conchotome approach [7]. Participants were not fasted during the blood sampling period, but given a standardised light meal (sandwich, crisps, yoghurt and drink) at the mid-point of the blood collections. Figure 1 (taken from [5] illustrates the protocol.

### Muscle functional tests

MVC was measured as previously described [2] with isometric contractions conducted in a sitting position using an isokinetic dynamometer at a 70° knee joint angle (Isocom Iskokinetics, Inc. De Queen, AR, USA). Participants were seated in the dynamometer chair and secured into position using a chest strap. Contractions lasted 5 s, with a 30-s rest period between contractions. Unilateral leg extension 1-RM was assessed on the dominant leg (ISO Leg Extension, Leisure Lines, Leicestershire, UK). After the procedure was explained, subjects performed a light warm up to ensure familiarity whilst avoiding fatigue; 1-RM was then achieved in as few repetitions as possible, with a maximum of 5 repetitions. The first repetition was estimated at 50% 1-RM and then increased until participants could not complete controlled contraction throughout the range of motion, with 3-min in-between attempts. Handgrip strength was assessed using a digital handgrip dynamometer (Grip D 5401; Takei Scientific Instruments Ltd, Japan) in each arm. The width of the dynamometer was adjusted for each participant, with participants instructed to stand upright with the arm fully extended along the body, maintaining an ∼5-cm gap between the wrist and the hip or upper leg so that the hand was not rested against the body. Participants were then instructed to squeeze against the handle as hard possible for 3 s. Grip strength was measured three times and recorded in kilogrammes to the nearest 0.1 kg. For the purpose of this study, the best of three attempts was included in further analysis from the stronger arm.

### Muscle architecture, DXA-derived mass

Muscle architecture was measured as described previously [2]. Briefly, the architectural parameter muscle thickness (MT), fascicle length (L*f*) and pennation angle (*θ*) were quantified, by the same investigator from ultrasound scans using ImageJ 1.42q software (National Institutes of Health, Bethesda, MD). The visible portion of the L*f* was assessed directly using this software with MT measured as the distance between the superficial and deep aponeuroses. *θ* was measured at the intersection between fascicles and the deep tendon aponeurosis. DXA-derived thigh fat-free mass (TFFM) was determined from the lowest point of the ischium to the mid-point of the patella.

### Statistical analyses

Here we aimed to assess whether any of the metabolic variables (MPS, MPB, D3-creatine, ASR) determined from the COSIAM approach, either individually or in combination with each other, could accurately predict our physiological outcomes (Handgrip, MVC, 1-RM, WBMM). Multivariate linear regression was performed using base R (https://cran.r-project.org/), with best subset regression implemented using the leaps package (https://cran.r-project.org/web/packages/leaps/index.html). Performance of each model was assessed using a combination of root mean squared error (RMSE) and *R* squared (*R*^2^) using the performance R Package (https://CRAN.R-project.org/package=performance), with those variables providing the lowest RMSE and highest *R*^2^ deemed to provide the best predictive power. All processing was performed in R (with scripts available from the authors upon request), and all plots were constructed in R using ggplot2 or GraphPad Prism v.9 (La Jolla, CA, USA). To assess sex differences in MPS and MPB, unpaired *t*-tests were performed following assessment for normal distribution of the data, and correlations were assessed using Pearson’s product moment correlation coefficient. The alpha level of significance for all measures was set at *p* < 0.05.

## Results

### Muscle protein synthesis (MPS), muscle protein breakdown (MPB) and muscle mass

**Fig. 2:**
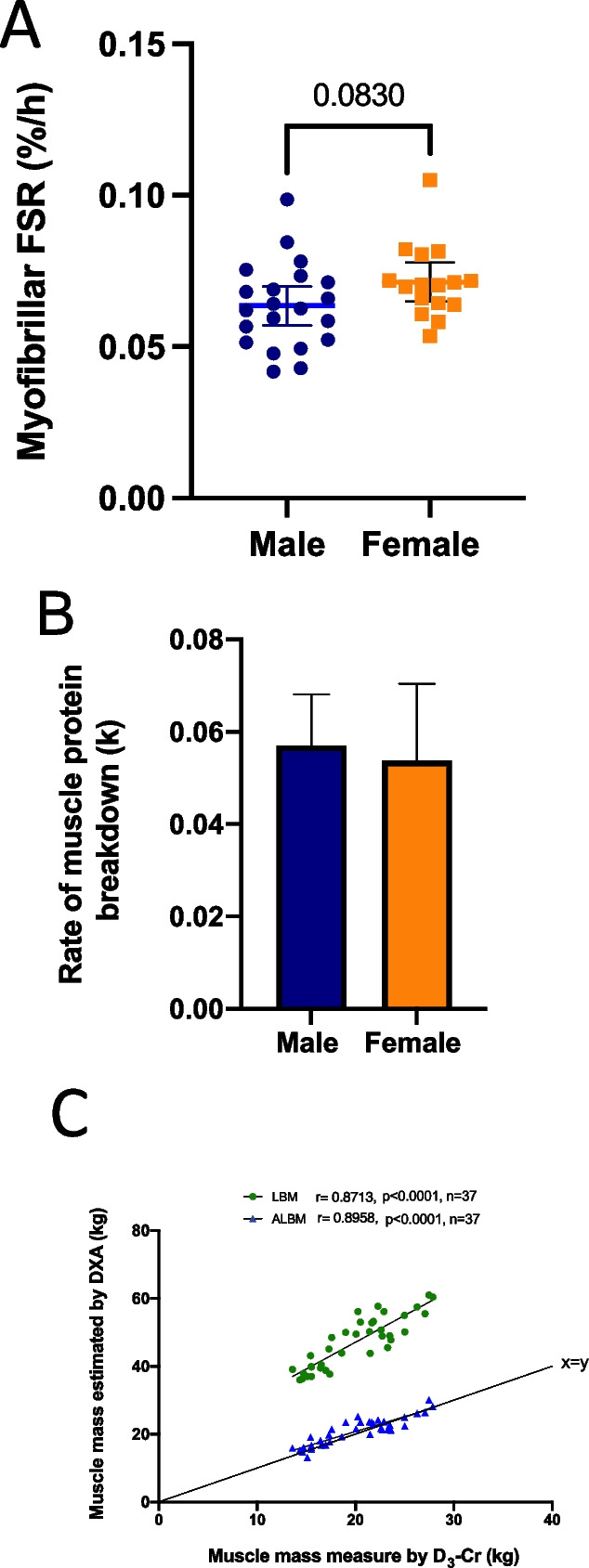
Sex differences for all COSIAM facets in older adults (*n* = 21 men, 16 women). **A** Muscle protein synthesis (MPS) as measured using D_2_O, **B** muscle protein breakdown (MPB) as measured via tracer dilution (k) rate of D_3_-3-Methylhistidine and **C** muscle mass as estimated using the D_3_-creatine dilution approach. Abbreviations: FSR, fractional synthetic rate; DXA, dual-energy absorptiometry

Mean MPS and MPB values as measured using the COSIAM approach are depicted in Fig. 2A and B. There were no differences observed between sexes, and measured rates fell within expected values for each, thereby confirming the accuracy and reproducibility of the COSIAM approach. Muscle mass as measured using D_3_-creatine showed significant positive correlations with both LBM and ALBM (Fig. 2C), with good agreement observed in absolute values between D_3_-creatine and ALBM.

### COSIAM as a measure of muscle health

**Fig. 3:**
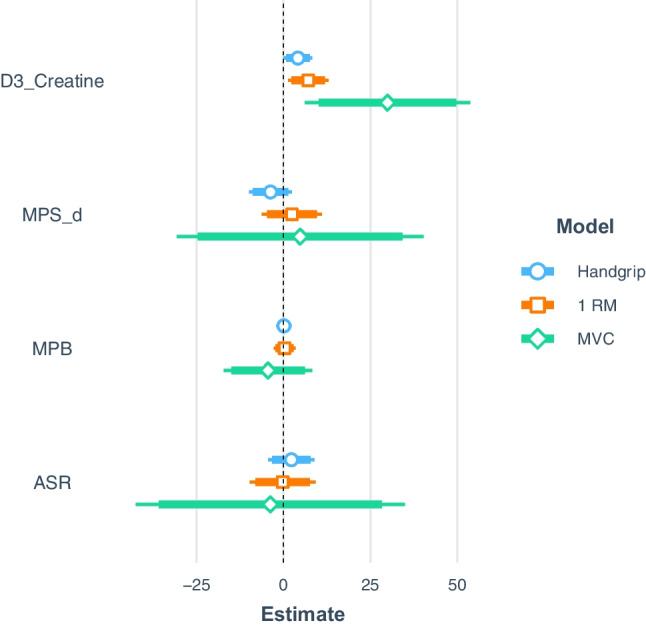
Forest plots showing the magnitude of effects for each predictor variable: MPS, MPB, ASR and D_3_-creatine, on each muscle health outcome variable (*n* = 29): handgrip strength, 1-RM and MVC, when each predictor variable is included in the regression model. Abbreviations: MPS-d, muscle protein synthesis per day; MPB, muscle protein breakdown; ASR, absolute synthetic rate; 1-RM, one-repetition maximum; MVC, maximum voluntary contraction

To assess the relationship between facets of the COSIAM approach (MPS, MPB, ASR and D_3_-creatine) and established measures of muscle health (handgrip strength, 1-RM and MVC), multivariate linear regression was used. Due to missing data for the key measures of 1-RM and MVC (as a result of unavailable equipment) for a small number of participants, linear regression was only possible to perform for *n* = 29. The aim of this approach was to assess which variable or group of variables provided the most optimal predictive model for each of the individual physiological measure of muscle health. This was performed using a stepwise or best subset regression approach implemented in the leaps R package, whereby a separate least squares regression model was fitted to each combination of the predictor variables: MPS, MPB, ASR and D_3_-creatine. To determine the performance of each of the models for predicting each of our outcome variables, k-fold cross validation was performed for each subset regression model, with the one providing the lowest prediction error determined to be the best predictive model. The magnitude of effect for each predictor variable in the full model is shown in Fig. 3. Using the best subset approach, D_3_-creatine alone was found to be the best predictor for each of the physiological measures of handgrip, 1R-M and MVC (see Table 1); the effect plots for each optimal model can be observed in Fig. 3, with D_3_-creatine showing a strong positive relationship with each outcome variable.

**Table 1:** Details of final best subset regression models for predicting each outcome variable. Abbreviations: *1 RM*, one-repetition maximum; *MVC*, maximum voluntary contraction; *nobs*, number of non-missing observations; *AIC*, Akaike information criteria; *BIC*, Bayesian information criteria

	Handgrip	1 RM	MVC
(Intercept)	6.05 (5.15)	− 3.16 (7.11)	2.02 (28.32)
D_3_-creatine	1.34*** (0.25)	1.64*** (0.35)	6.53*** (1.38)
nobs	29	29	29
*r* squared	0.51	0.45	0.45
Adj. *r* squared	0.50	0.43	0.43
Sigma	5.52	7.62	30.37
Statistic	28.64	22.48	22.50
*p* value	0.00	0.00	0.00
df	1.00	1.00	1.00
logLik	− 89.66	− 99.00	− 139.10
AIC	185.32	204.01	284.21
BIC	189.42	208.11	288.31
Deviance	822.86	1567.47	24,902.60
df. residual	27.00	27.00	27.00
nobs. 1	29.00	29.00	29.00

^***^p < 0.001; **p < 0.01; *p < 0.05

D_3_-creatine as highlighted in Fig. 2C has a strong relationship with DXA-derived WBMM. Therefore, to determine whether D_3_-creatine performed as a better predictor of each physiological outcome than DXA-derived measures, linear regression models of D_3_-creatine and DXA-derived lean mass were constructed and predictive performance of both models directly compared. Performance metrics for each model are provided in Table 2. For all three physiological outcomes of handgrip, 1RM and MVC, D3-creatine performed better at predicting outcomes than DXA-derived lean mass, as evidenced by the greater *R* squared (and adjusted *R* squared) and lower RMSE for D_3_-creatine.

**Table 2:** Performance metrics for D_3_-creatine and DXA-derived lean body mass regression models. Abbreviations: *DXA*, dual-energy x-ray absorptiometry; *1 RM*, one-repetition maximum; *MVC*, maximum voluntary contraction; *RMSE*, root-mean-square deviation; *AIC wt*, Akaike information criteria weight

Model name	*R*^2^	*R*^2^ adjusted	RMSE	Sigma	AIC wt	Performance score
Handgrip ~ D3_Creatine	0.514	0.496	5.326	5.520	0.678	1
Handgrip ~ DXA	0.489	0.470	5.465	5.664	0.322	0
1 RM ~ D3_Creatine	0.454	0.434	7.351	7.619	0.94	1
1 RM ~ DXA	0.339	0.315	8.088	8.382	0.059	0
MVC ~ D3_Creatine	0.454	0.434	29.304	30.370	0.922	1
MVC ~ DXA	0.35330066	0.329	31.909	33.069	0.078	0

## Discussion

The present study follows on from prior pilot work in which we developed a protocol fit for purpose to simultaneously quantify WBMM, MPS and MPB using the COSIAM approach. In the face of increasing difficulties in deploying alternative techniques to quantify these facets of muscle health (outlined below), we sought to develop a more practicable, less invasive and more widely applicable method. For example, prohibitively expensive imaging methods such as MRI are often use for WBMM whilst stable isotopically labelled amino acids by I.V infusions for quantifying MPS are limited by their short-term nature, need for pharmacy QC, costs and ethical issues. Similarly quantifying MPB is a notoriously challenging dynamic measurement where A-V balance models, for instance, rely on the detection of negligible tracer dilution and accurate blood flow measurements across a limb bed.

The COSIAM approach that we developed [5] indicated accurate measures of diurnal MPS and MPB in accordance with our and others’ prior research [9, 19]. In terms of sex, despite no statistical differences, women tended to exhibit numerically higher rates of MPS than older men. Differences in acute (i.e. over a few hours) MPS were previously reported between older men and women [15], with it being proposed that a greater *basal* (i.e. postabsorptive) MPS which operates over a majority of the diurnal cycle may explain slower age-related muscle loss in women than in men. Our present results in showing a trend (but not statistical significance) underline at this stage, if nothing else, potential sexual dimorphism of protein metabolism in ageing and the need to consider sex differences in the wider field of age-related sarcopenia as necessary. Also, that integrated measures of MPS over day(s) may not reflect those of a single diurnal fasted-fed cycle. Finally, our work is also in agreement with others showing that whilst D_3_-Cr measures of muscle mass correlate with DXA, DXA markedly underestimates WBMM, which is intuitive given that a major limitation of DXA is its inability to distinguish between lean tissue and muscle tissue per se [13].

**Fig. 4:**
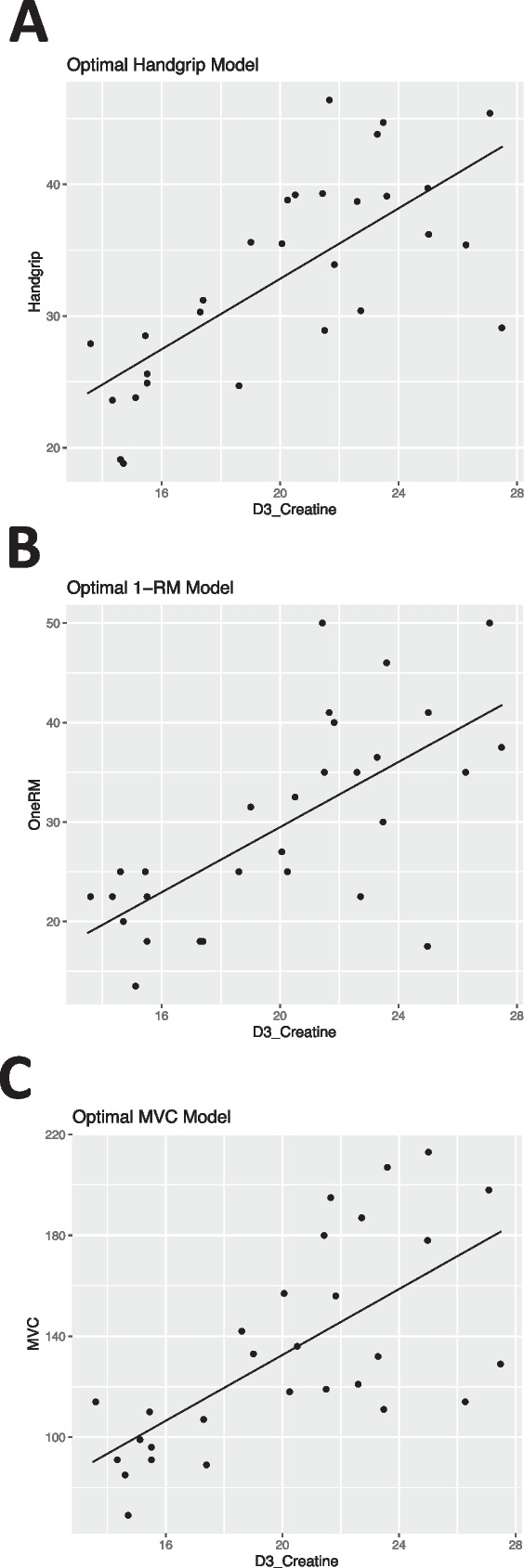
Effect plots for each of the optimised best subset models highlighting the positive linear relationships between D_3_-creatine and **A** handgrip strength, **B** one-repetition maximum (1-RM) and **C** maximum voluntary contraction (MVC, *n* = 29)

The principal objective of this study was to determine links between facets of COSIAM and that of more established biomarkers of muscle mass and function. To address this, rather than focusing upon myriad individual correlations, we undertook multivariate linear regression to assess which variable—or groups of variables—provided the most optimal model for predicting each individual physiological measure of muscle vitality. In this cross-sectional trial, we report that D_3_-Cr-derived WBMM was able to best predict core markers of muscle health, including handgrip strength (used in much epidemiology muscle research [11]) and knee-extensor 1-RM and MVC, commonly used as muscle strength assessments in muscle physiology circles. The notion of D_3_-Cr-derived WBMM being superior to other imaging techniques has been established, with low D_3_-Cr-derived muscle mass being identified as a novel risk factor for clinically meaningful outcomes in older men [4], and with associations of D_3_-Cr being more closely associated with measures of physical function in older women, than DXA [22]. Our work corroborates that even when compared to MPS and MPB, cross-sectionally D_3_-Cr-derived WBMM most closely relates to muscle function. It is likely that this is due to D_3_-Cr providing a direct readout of contractile mass (Fig. 4) .

**Fig. 5:**
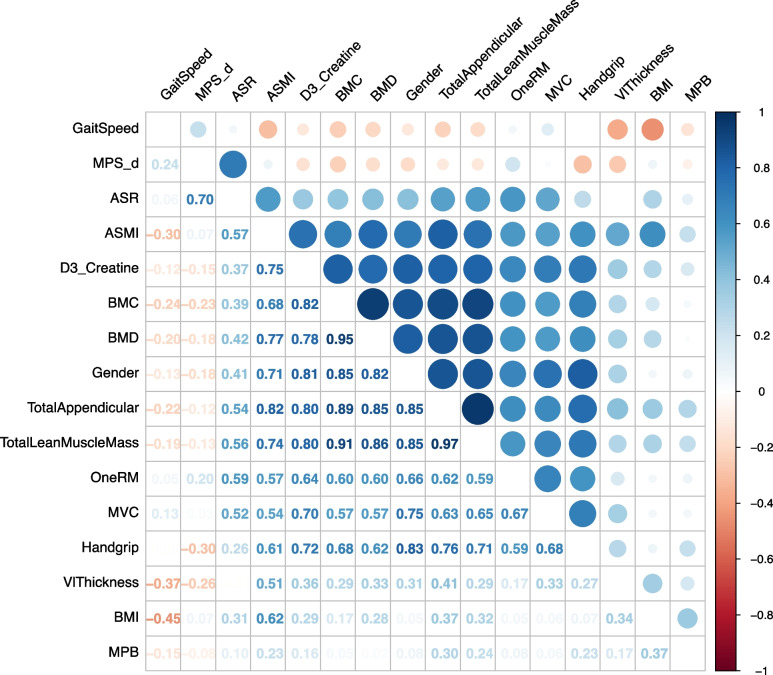
Correlation matrix of relationships amongst all measured variables (*n* = 29). Abbreviations: MPS-d, muscle protein synthesis per day; ASR, absolute synthetics rate; ASMI, appendicular skeletal mass index; BMC, bone mineral content; BMD, bone mineral density; RM, repetition maximum; MVC, maximum voluntary contraction; Vl, vastus lateralis; BMI, body mass index; MPB, muscle protein breakdown

We also provide readers with a matrix of relationships between each of the variables that were measured in this trial (Fig. 5). This matrix illustrates the strengths of some of these already well-described links between variables, for instance, between handgrip strength and sex, but also more novel observations such as those between MPS rates corrected for muscle mass (absolute synthesis rate (ASR)), and measures of muscle functional capacity such as 1-RM and MVC. This may suggest a potential application of measuring MPS for predicting functional outcomes when corrected for muscle mass; however, further validatory work is needed to confirm this. Finally, this correlation matrix further highlights the strength of relationships between D_3_-Cr-derived WBMM and DXA/muscle function outcomes, and hence its power for potentially predicting these outcomes as evidenced by this work.

Finally, we acknowledge potential limitations of COSIAM, which include the whole body (non-regional) nature of the measure of muscle mass,; that assumptions are based on a standardised creatine pool size (although with a muscle biopsy, direct creatine measures can be made in a given population); that whilst somewhat minimally invasive, this approach as of now still requires a muscle biopsy; and that diet and exercise behaviours could impact outcomes and could/should be monitored to assess any study- or population-specific effects, in future. Despite this, the COSIAM method is a minimally invasive and valid method to assess WBMM, MPS and MPB simultaneously, offering much potential in clinical and difficult to study populations, e.g. care home residents and patient populations at risk of muscle wasting. Whilst the present work cements D_3_-Cr as a robust cross-sectional biomarker of muscle physiology, future *interventional* work will establish how protein turnover interacts with D_3_-Cr-derived measures of WBMM in situations of change, e.g. during acute illness, chronic disease or in response to exercise/drug or other interventions.
